# Dynamic oropharyngeal and faecal microbiota during treatment in infants hospitalized for bronchiolitis compared with age-matched healthy subjects

**DOI:** 10.1038/s41598-017-11311-z

**Published:** 2017-09-12

**Authors:** Qian Hu, Wenkui Dai, Qian Zhou, Dan Fu, Yuejie Zheng, Wenjian Wang, Yanhong Liu, Qin Yang, Dongling Dai, Sixi Liu, Guosheng Liu, Shuaicheng Li, Feiqiu Wen

**Affiliations:** 1 Department of Hematology and Oncology, Shenzhen Children’s Hospital, Shenzhen, 518038 China; 2 Department of Computer Science, City University of Hong Kong, 999077 Hong Kong, China; 3 Department of Respiratory Diseases, Shenzhen Children’s Hospital, Shenzhen, 518038 China; 4 Department of Microbial Research, WeHealthGene, Shenzhen, 518000 China; 5 Department of Emergency, Shenzhen Children’s Hospital, Shenzhen, 518038 China; 6 Department of Digestive Diseases, Shenzhen Children’s Hospital, Shenzhen, 518038 China; 7 Division of Neonatology, The Affiliated 1st Hospital, Jinan University, Guangzhou, 510632 China

## Abstract

Bronchiolitis is one of the most severe diseases affecting infants worldwide. An imbalanced oropharynx (OP) microbiota has been reported in infants hospitalized with bronchiolitis; however, the microbiota dynamics in the OP and faeces during therapy remain unexplored. In total, 27 infants who were hospitalized with bronchiolitis were selected for this study, and sampling was conducted before therapy and after clinical recovery. We also recruited 22 age-matched healthy infants for this study. The faecal and OP microbiota diversity in the patients was lower than that in the healthy children. The faecal microbiota (FM) in the diseased children significantly differed from that in the healthy subjects and contained accumulated *Bacteroides* and *Streptococcus*. The OP microbiota in both the healthy and diseased infants was dominated by *Streptococcus*. After the treatment, the FM and OP microbiota in the patients was comparable to that before the treatment. This study may serve as an additional reference for future bronchiolitis studies, and the “risk microbiota model” of clinically recovered infants suggests an increased susceptibility to pathogen intrusion.

## Introduction

Bronchiolitis is the leading cause of hospitalization of infants worldwide and is characterized by rapid spreading of upper respiratory tract (URT) infections to the lower respiratory tract (LRT)^1^. Respiratory syncytial virus (RSV) is the main agent causing bronchiolitis in infants^2, 3^. Rhinovirus, bocavirus, and human metapneumovirus infections are also commonly observed in paediatric bronchiolitis^2, 3^.

Common pathogens, including viruses and bacteria, can be identified through conventional culturing or PCR amplification^4^. However, these pathogens represent a small fraction of the microbial organisms living in the gastrointestinal and respiratory tracts^5, 6^. Ubiquitous opportunistic pathogens are present in the URT, but not all infants develop respiratory diseases^7–10^. Opportunistic pathogens are also commonly present in the intestine during infancy. These pathogens foster stable homeostasis with other microbial commensals^11–13^. Therefore, a stable microbial structure may be important for the prevention of infection and disease.

Several studies have explored imbalances in the faecal and nasopharyngeal microbiota during the onset of bronchiolitis, which has improved the understanding of the micro-ecology of bronchiolitis^14–17^. Carlos A. Camargo Jr. *et al*. performed a retrospective study involving 1,005 infants hospitalized for bronchiolitis^14, 15^. In total, 4 nasopharyngeal microbiota profiles were identified; of these profiles, the *Haemophilus*-dominated profile was closely associated with intensive care use (ICU) and a prolonged hospitalization time^14, 15^. This finding was also confirmed in an additional 307 infants hospitalized for bronchiolitis^14^. The microbiota profile was associated with the disease severity only in infants with low serum cathelicidin (LL-37 ≤46 ng/ml)^15^. Five additional nasopharyngeal microbiota clusters enriched with *Haemophilus influenza*e, *Streptococcus*, *Corynebacterium*, *Moraxella*, and *Staphylococcus aureus* have been identified^16^. The *H. influenzae-* and *Streptococcus*-dominant profiles were positively associated with RSV infections, host expression of toll-like receptors, and neutrophil and macrophage activation and signalling^16^. Carlos A. Camargo Jr. *et al*. also conducted a study investigating the faecal microbiota (FM) in 40 infants hospitalized for bronchiolitis and 115 healthy infants^17^. The infants with the *Enterobacter/Veillonella*-dominant microbiota cluster exhibited the lowest incidence of bronchiolitis, and the *Bacteroides*-dominant microbiota profile was associated with the greatest incidence of bronchiolitis onset^17^. The *Escherichia*- and *Bifidobacterium*-enriched microbiota clusters were associated with a medium incidence^17^. A longitudinal study involving 265 neonates with healthy records was performed until the participants were 3 years of age^18^. The infants who harboured positive cultures of *Streptococcus pneumoniae*, *H. influenzae*, and *Moraxella catarrhalis* in their hypopharynx at four weeks of age had a higher incidence of pneumonia and bronchiolitis^18^.

The oropharynx (OP) microbiota is more analogous to the LRT microbiota than to the NP microbiota^19, 20^. However, few reports have evaluated the OP microbiota in patients with bronchiolitis. Furthermore, the dynamics of the OP microbiota and FM during clinical therapy remain unclear, and such knowledge could improve the understanding of repeated infections in paediatric bronchiolitis. In this study, 27 infants who were hospitalized for bronchiolitis and 22 age-matched healthy infants were recruited from Shenzhen Children’s Hospital, and a comparative analysis of the FM and OP microbiota was performed. We explored the following two issues in this study: 1. whether the FM and OP microbiota in the diseased children differed from those in the healthy infants and 2. whether the FM and OP microbiota structures changed after clinical therapy.

## Results

### Participants and data output

We selected 27 infants who were hospitalized for mild bronchiolitis from Shenzhen Children’s Hospital (Table 1, Supplementary Table); all infants were diagnosed with a human RSV infection. The primary medical treatment during hospitalization was nebulized budesonide combined with salbutamol (Supplementary Table). Two bacterial pathogens, i.e., *Streptococcus pneumonia* and *Haemophilus parainfluenzae*, were identified in bacterial cultures of sputum from 5 hospitalized infants (Supplementary Table). Based on clinical experience or diagnoses of bacterial pathogens, 11 diseased infants received antibiotic treatment (Supplementary Table). No inpatient was admitted to the paediatric intensive care unit (PICU) or given mechanical ventilation during hospitalization. Most hospitalized infants remained in the hospital for 3–10 days, except for the following two infants: one infant stayed in the hospital for 13 days, and another infant stayed in the hospital for 26 days (Supplementary Table). The longer hospital stays of these two inpatients were due to secondary infections with rotavirus after clinical remission of bronchiolitis. In addition, 22 age-matched healthy infants were recruited in Shenzhen, China (Table 1, Supplementary Table). In total, 4,898,610 tags were obtained using 16S rDNA amplicon sequencing, ranging from 10,617 to 55,487 tags per sample.

**Table 1:** Sample information.

	Patients (n = 27)	Healthy (n = 22)
Demographic characteristics		
Age (months)	4.3 (1.3–11)	6.1 (1.4–10.7)
Male	18 (66.7%)	7 (31.8%)
Breast feeding	16 (59.3%)	12 (54.5%)
Premature	2 (0.07%)	5 (22.7%)
C-section	11 (40.7%)	11 (50%)
History of eczema	6 (22.2%)	1(4.5%)
Maternal asthma	4 (14%)	3(13.6%)
Maternal smoking	14 (51.9%)	4(18.2%)
Disease situation		
Hospitalization time (days)	7 (3–26)	NA
Fever	14 (51.9%)	NA
Wheezing	18 (66.7%)	NA
Dyspnea	3 (11.1%)	NA
Three concave sign	5 (18.5%)	NA
Anhelation	13 (48.1%)	NA
Cyanosis	5 (18.5%)	NA
Moist rales	17 (63%)	NA
Increase of lung markings	21 (77.8%)	NA
Patch shadow	12 (44.4%)	NA
Eosnophils (0.5–5%)	20 (74.1%)	NA
CRP (<0.499)	13 (48.1%)	NA

### Confounder analysis

Bronchiolitis onset, age, body weight, gender, delivery mode, feeding pattern, history of eczema, maternal asthma and smoking status were selected for the analysis of the main contributing factors to the inter-group discrepancies. According to the association analysis, the bronchiolitis onset significantly contributed to the differences in the FM/OP microbiota between the healthy and diseased children (*q-*value < 0.001, Supplementary Table).

### FM and OP microbiota of the infants with bronchiolitis differed from that of the healthy infants

**Figure 1:**
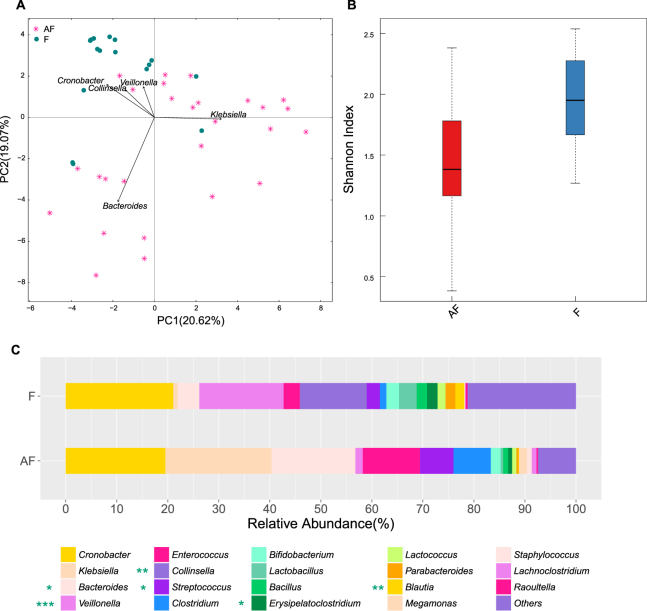
Comparison of the FM in the patients and healthy infants. AF: faecal samples collected from diseased children within 24 h of hospitalization. F: faecal samples collected from the healthy infants. (**A**) Pink plots represent the patients, and green circles represent the healthy infants. (**B**) Boxplot of the alpha diversity in the FM. (**C**) Stacked bar of the relative abundance at the genus level. *, ** and *** noted next to the genus represent *q*-values ≤0.05, ≤0.01 and ≤0.001, respectively.

The FM structure in the hospitalized infants differed from that in the age-matched healthy infants (Fig. 1-A). The FM in the diseased infants exhibited a lower diversity than that in the healthy infants (Fig. 1-B). Firmicutes Bacteroidetes, Proteobacteria and Actinobacteria accounted for >99% of the FM in both the hospitalized and healthy infants (Supplementary Table), and Proteobacteria (42.14%) and Bacteroidetes (17.31%) were enriched in the FM in the hospitalized infants (Supplementary Table). At the genus level, the diseased infants harboured more *Bacteroides* (16.44% vs 4.26% in the healthy infants, *q*-value < 0.05) and *Streptococcus* (6.54% vs 2.60% in the healthy infants, *q*-value < 0.05) in the FM (Fig. 1-C, Supplementary Table). *Klebsiella* (20.80% vs 0.86% in the healthy infants)*, Clostridium* (7.32% vs 1.27% in the healthy infants) and *Enterococcus* (11.20% vs 3.19% in the healthy infants) were also more prevalent in the FM in the children with bronchiolitis, but this difference was not statistically significant (Fig. 1-C, Supplementary Table). By contrast, *Collinsella* (0.03% vs 13.09% in the healthy infants, *q*-value < 0.01), *Veillonella* (1.46% vs 16.51% in the healthy infants, *q*-value < 0.001), *Blautia* (0.08% vs 1.78% in the healthy infants, *q-value* < *0.01*), and *Erysipelatoclostridium* (0.77% vs 2.04% in the healthy infants, *q*-value < 0.05) accumulated in the FM in the hospitalized infants (Fig. 1-C, Supplementary Table).

**Figure 2:**
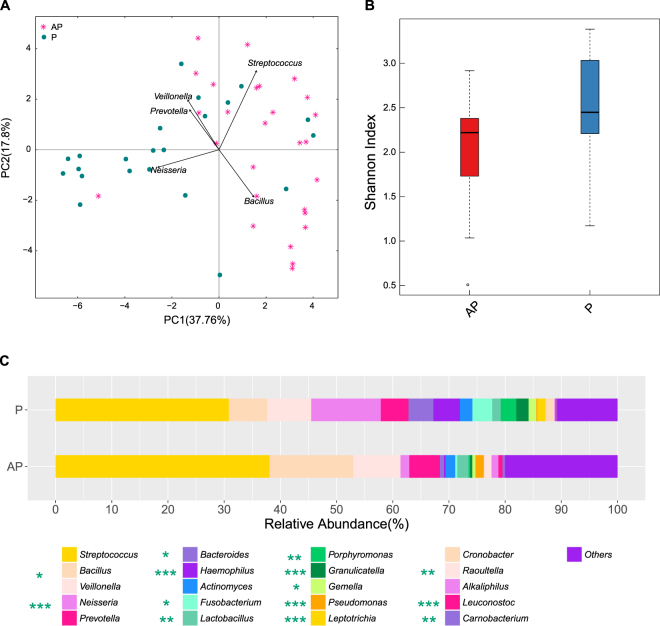
Comparison of OP microbiota in the patients and healthy controls. AP: oropharyngeal swabs sampled from diseased children over the course of 24 h of hospitalization. P: oropharyngeal swabs sampled from healthy infants. (**A**) Pink plots represent the patients, and green circles represent the healthy infants. (**B**) Boxplot of the alpha diversity in the OP microbiota. (**C**) Stacked bar of the relative abundance at the genus level. *, ** and *** noted next to the genus represent *q*-values ≤ 0.05, ≤ 0.01 and ≤ 0.001, respectively.

Similar to the FM, the OP microbial structure also differed between the healthy and diseased infants (Fig. 2-A), and the OP microbiota diversity in the hospitalized infants was lower than that in the healthy infants (Fig. 2-B). Firmicutes (84.79%, *q-*value < 0.01) was dominant in the diseased infants, while Bacteroidetes (13.94%, *q*-value < 0.01) and Proteobacteria (20.46%, *q*-value < 0.05) accumulated dramatically in the healthy children (Supplementary Table). The OP microbiota in both the healthy and hospitalized infants was dominated by *Streptococcus* (Fig. 2-C, Supplementary Table). Several genera accounted for the lowered abundance of the OP microbiota in the hospitalized infants, including *Neisseria* (1.57% vs 12.40% in the healthy infants, *q*-value < 0.001), *Bacteroides* (0.71% vs 4.43% in the healthy infants, *q*-value < 0.05), *Haemophilus* (0.28% vs 4.72% in the healthy infants, *q*-value < 0.001), *Granulicatella* (0.40% vs 2.19% in the healthy infants, *q*-value < 0.001), *Leptotrichia* (0.07% vs 1.46% in the healthy infants, *q*-value < 0.001), and *Porphyromonas* (0.29% vs 2.78% in the healthy infants, *q*-value < 0.01) (Fig. 2-C, Supplementary Table). By contrast, *Bacillus* (14.90% vs 6.86% in the healthy infants, *q*-value < 0.05), *Pseudomonas* (1.44% vs 0.19% in the healthy infants, *q*-value < 0.001), and *Raoultella* (1.28% vs 0.16% in the healthy infants, *q*-value < 0.01) accumulated in the OP microbiota in the diseased infants (Fig. 2-C, Supplementary Table).

### Treatment-induced changes in the FM/OP microbiota were few and individual-specific

**Figure 3:**
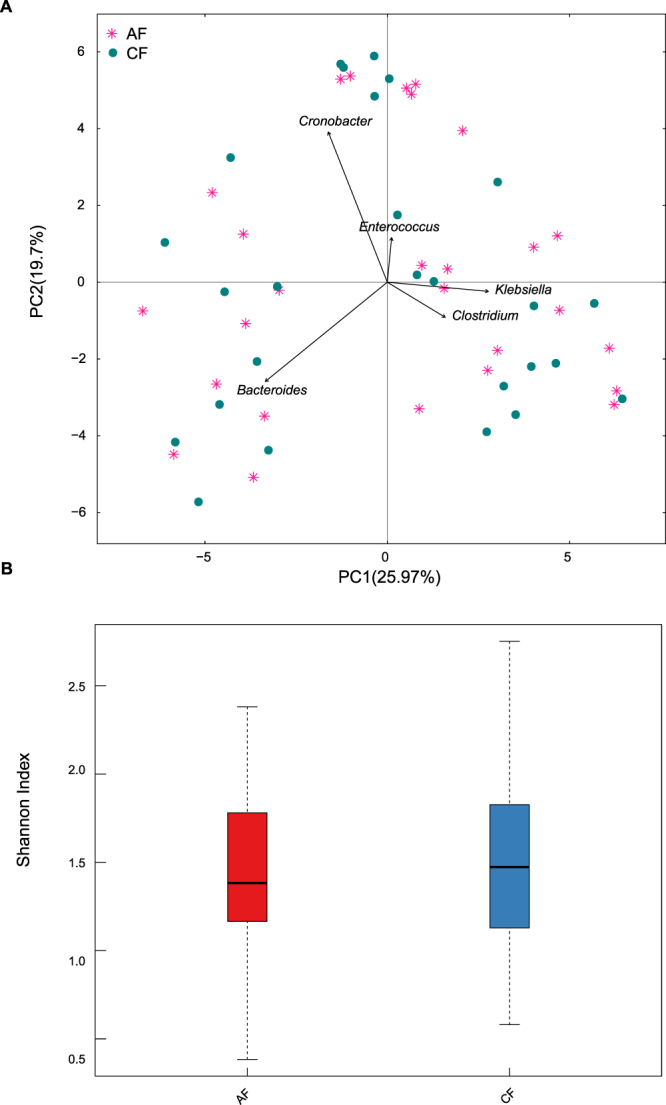
Comparison of the FM at two sampling time points in the patients. AF: within 24 h of hospitalization. CF: clinical recovery. (**A**) Pink plots represent the AF, and green circles represent the CF. (**B**) Boxplot of the alpha diversity in the FM.

**Figure 4:**
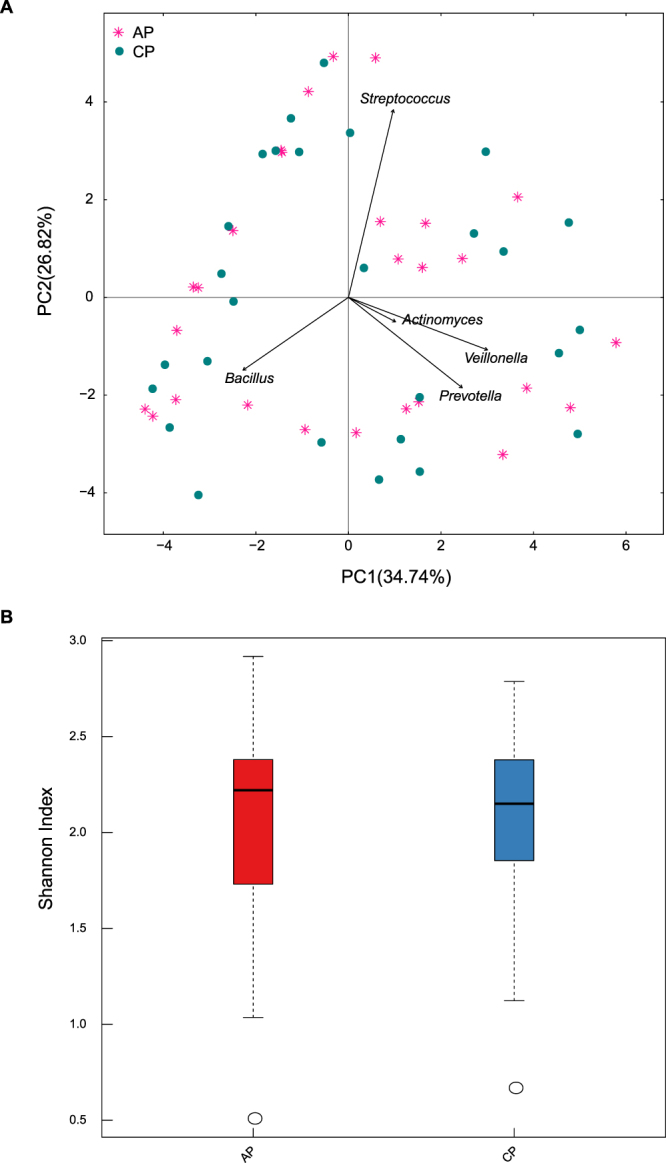
Comparison of the OP microbiota at two sampling time points in the patients. AP: within 24 h of hospitalization. CP: clinical recovery. (**A**) Pink plots represent the AP, and green circles represent the CP. (**B**) Boxplot of the alpha diversity in the OP microbiota.

The FM and OP microbiota structures of clinically recovered patients were similar to that before therapy(Figs 3–4). A comparative analysis of each case was also conducted to understand the microbiota changes in each hospitalized infant (Supplementary Figures 1–2).

*Haemophilus influenzae* or *Streptococcus pneumoniae* were identified in sputum cultures from 5 patients, whose FM diversity was similar to that of the other patients (Supplementary Figure 1A, Supplementary Table). Only two diseased children (patients 6 and 7) received oral antibiotics (Supplementary Table), and their FM diversity significantly decreased after therapy (Supplementary Figure 1B). By contrast, a comparable or higher FM diversity was identified in the other patients (Supplementary Figure 1A) who received intravenous or no antibiotic therapy (Supplementary Table). *Klebsiella, Enterococcus, Bacteroides, Cronobacter* and/or *Clostridium* dominated the FM in the diseased infants and did not change after the different therapies (Supplementary Figure 1B). After clinical therapy, the OP microbiota diversity in the hospitalized infants increased or remained the same, even in patients who received oral antibiotics (Supplementary Figure 2A). *Streptococcus* remained dominant in the OP microbiota in the diseased infants during therapy (Supplementary Figure 2B). *Bacillus*, *Veillonella*, *Prevotella* and *Raoultella* also dominated after treatment (Supplementary Figure 2B).

## Discussion

Bronchiolitis is mainly caused by viral infections and co-infections with bacterial pathogens^2, 3^. Viral and bacterial infections have been shown to induce an immune response and microbiota imbalance^21^. This study revealed an imbalanced FM/OP microbiota in children with viral bronchiolitis, which is consistent with that identified in other respiratory diseases, such as asthma and cystic fibrosis^22, 23^.

The distal effect of the gut microbiota (GM) on respiratory health is profoundly affected by the regulation of the host immune system^23^. The GM plays a crucial role in the immune response to and protection from pulmonary infection^24–28^. *Bifidobacterium* and *Lactobacillus* tend to accumulate in healthy infants but not in significant amounts. *Bifidobacterium* spp. was found to be protective against both bacterial and viral infections of the respiratory tract^27, 29, 30^. Probiotics composed of *Bifidobacterium* and *Lactobacillus* are also promising for the control of respiratory infections^31–34^. In the healthy children, enriched *Veillonella* was positively associated with Th17-mediated immunity in the lungs^35^ and negatively associated with the risk of asthma^36^. *Bacteroides* and *Streptococcus* accumulated in significant amounts and represented a high proportion of FM in diseased children. Several members of *Streptococcus* spp. could induce inflammation in the epithelial mucus^37^, including the insignificantly enriched *Klebsiella* in diseased infants^38^. By contrast, various *Bacteroides* spp. have the potential to relieve inflammation by expanding the T_reg_ cell population and suppressing inflammatory responses^23^. The aforementioned prior study could partially explain the GM imbalance observed in the diseased children. In all patients, except for patients 6 and 7, who took oral antibiotics, the FM diversity either remained unchanged or decreased after the treatment. This finding indicated the need to decrease exposure to oral antibiotics, which could block GM recovery for several months^39^.

Numerous reports have described the OP microbiota in healthy children, which was primarily dominated by *Streptococcus*, *Rothia*, *Prevotella*, *Gemella*, *Veillonella*, *Fusobacteria*, *Haemophilus*, and *Neisseria* ^4^. Nader Shaikh *et al*. also reported prevalent streptococcal carriage in children without pharyngitis symptoms^40^. We found microbial carriage in the OP in the healthy infants that was identical to that described in these reports^4, 40^. However, the OP microbial structures in the hospitalized infants significantly differed from those in the healthy infants, which potentially indicates transmission to the lung^19, 20^. Several reports have identified four microbiota profiles in the NP in infants hospitalized for bronchiolitis, and the *Haemophilus*-dominated profile was associated with the highest clinical severity^14, 15^. We established a *Streptococcus*-dominant OP microbiota profile in both the hospitalized and healthy infants, which could be attributed to the differing microbiota components between the NP and OP^19, 20^. Moreover, the OP microbiota remained unchanged in most patients who recovered clinically. This finding may explain the widespread repeated respiratory infections in children after therapy because high incidences of respiratory diseases have been reported in the presence of “unstable” URT microbiota^8, 41^. Therefore, the OP microbiota has promising potential in bronchiolitis prognosis, therapy optimization, and evaluation of recovery.

This study also had some limitations. A total of 47 infants was not sufficient for partitioning information according to discrepant clinical symptoms, such as that performed by Carlos A and Camargo Jr. *et al*.^14, 15^. The detailed clinical symptoms and host responses, including immune cytokines, should be considered to assess the contributions of microbiota. Long-term investigations exploring the incidence of acute respiratory infection in infants who recovered clinically should also be performed.

In conclusion, this study reviewed the imbalanced FM and OP microbiota in Chinese children with bronchiolitis and provided additional reference data for associated studies. More importantly, we identified comparable OP and FM microbiota among patients before and after treatment. This finding suggested a risk model of OP microbiota in children who recover clinically^42^.

## Materials and Methods

### Ethics statement

This study was approved by the Ethical Committee of Shenzhen Children’s Hospital under the registration number 2015020. All procedures were performed in accordance with the relevant guidelines and regulations stipulated by the Ethical Committee of Shenzhen Children’s Hospital. We obtained written informed consent from the parents of all participants, who approved their children’s participation in the study.

### Sample preparation

In total, 27 inpatients (aged ≤1 year of age) were sampled within 24 h of hospitalization (before therapy) in Shenzhen Children Hospital, and the second sampling was conducted after clinical recovery from bronchiolitis (average 7–10 days after treatment). In addition, 22 age-matched healthy infants were recruited according to the following inclusion criteria: no wheezing, fever, cough, or other respiratory/allergic symptoms at the time of sampling and for 2 weeks prior to the study and no respiratory symptoms for 1 week after sampling. None of the infants was exposed to antibiotics for two weeks before sampling. Sterile oropharyngeal swabs (155 C, COPAN, Murrieta, California, USA) were used for sampling the OP and faeces. For the diseased infants, we performed the sample collection during the following two time points: 24 hours after hospitalization and upon clinical recovery (average 7–10 days after treatment). The collected samples were immediately stored at −80 °C, and the DNA extraction was performed within one week. The sputum samples were collected by performing endotracheal suctioning (AARC (American Association for Respiratory Care) Clinical Practice Guidelines)^43^ and cultured for several bacterial pathogens.

### DNA extraction, sequencing, and analysis

DNA was extracted using a PowerSoil® DNA Isolation Kit (Mo Bio Laboratories) according to the manufacturer’s protocol. The hypervariable V3–V4 region of the 16S rRNA gene was amplified as previously reported^44^ and was sequenced using an Illumina MiSeq platform. The paired-end reads were filtered using Mothur’s Miseq SOP^45^ and connected to tags using FLASH (v1.2.11, http://ccb.jhu.edu/software/FLASH/index.shtml). The qualified tags were clustered into operational taxonomic units (OTUs), and OTUs from chimaeras were removed using USEARCH (v7.0.1090). The OTUs were assigned a taxonomic classification by aligning to the RDP 16S rRNA database (201408).

The multivariable analysis was conducted via a PERMANOVA (permutations = 9999, *P*-value ≤0.05) using the R package “vegan” (version 2.4-3)^46^ to identify the important factors that could be associated with the microbiota structure. The Wilcoxon rank-sum test was used to compare the inpatients to the healthy infants, and a time-series comparison of the hospitalized infants was performed using the Wilcoxon signed-rank test. *P*-values were adjusted according to the false discovery rate for multiple tests. *, ** and *** represent *q*-values ≤0.05, ≤0.01 and ≤0.001, respectively. Increases/decreases in the Shannon index of FM and OP microbiota diversity >50% were considered significant. R (version 3.2.3) and SVG (version 1.1) software packages were used for visualization.

### Accession number

Clean reads were deposited in the GenBank database under accession number PRJNA362484.

## Electronic supplementary material

Supplementary Figure

Supplementary Table
