# Supplementary material for: Dynamic oropharyngeal and faecal microbiota during treatment in infants hospitalized for bronchiolitis compared with age-matched healthy subjects
Source: Sci Rep. 2017 Sep 12;7:11266. doi: 10.1038/s41598-017-11311-z (PMC5595837; doi:10.1038/s41598-017-11311-z)

Title: Dynamic oropharyngeal and faecal microbiota during treatment in infants hospitalized for bronchiolitis compared with age-matched healthy subjects

Qian Hu[3,#], Wenkui Dai[2,#], Qian Zhou[4,#], Dan Fu[5], Yuejie Zheng[3], Wenjian Wang[3], Yanhong Liu[4], Qin Yang[3], Dongling Dai[6], Sixi Liu[1], Guosheng Liu[7], Shuaicheng Li[2], Feiqiu Wen[1,*]

Supplementary Figure 1. **Dynamic FM of pediatric patients during therapy**. **A:** AF and CF represent samples taken from diseased children before and after treatment. The 2nd sample was removed because lack of post-treatment data. Star signed below the patient number means Shannon index change >50%. **B:** The log10 value of relative abundance was calculated and described in different colors. The X-axis provides the samples' ids. The Y-axis shows accumulated genera at different stages (A: 24 h after hospitalization; C: clinically recovered)**.**

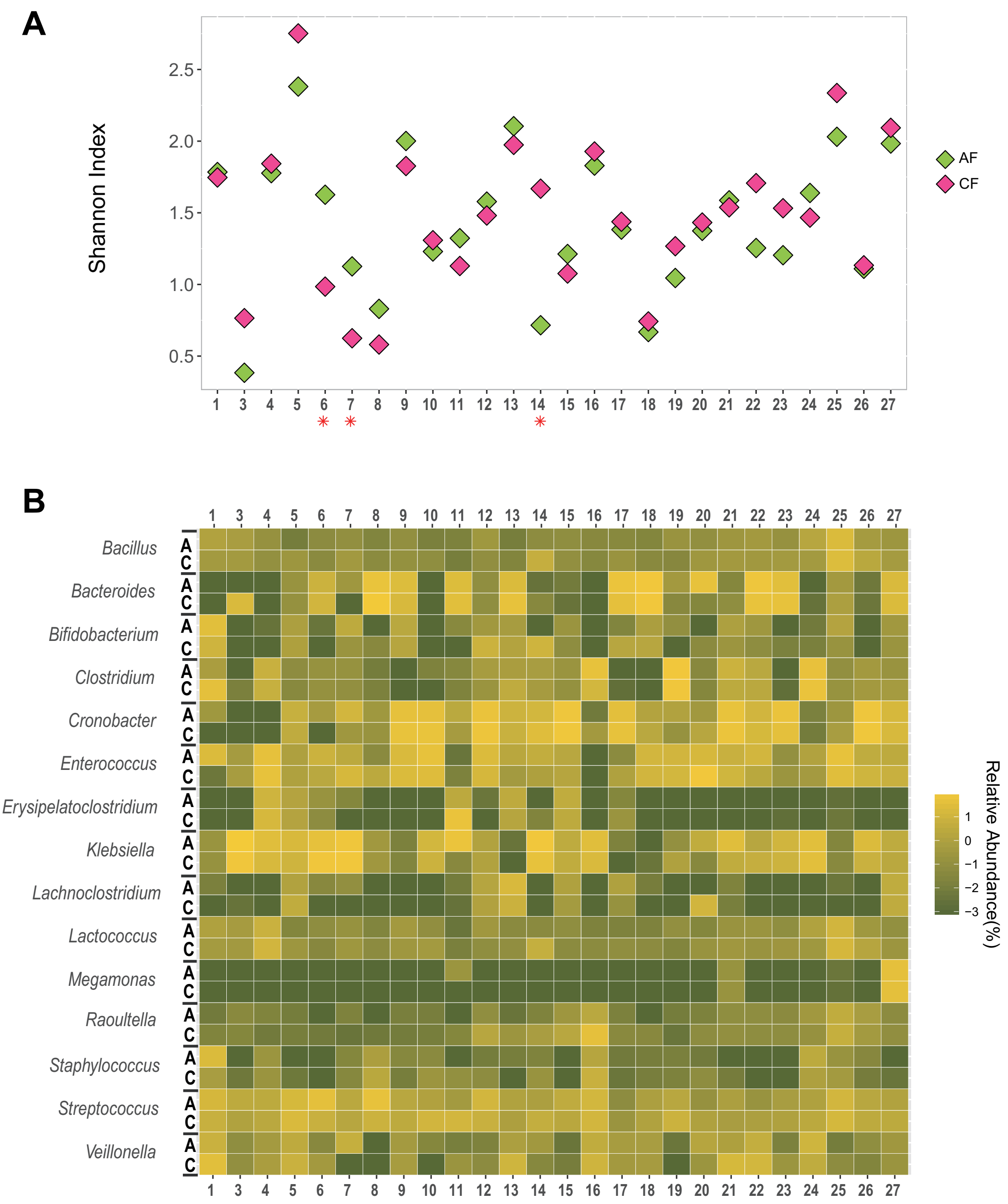

Supplementary Figure 2. Dynamic OP microbiota of pediatric patients during therapy. A: AP and CP represent samples taken from children before and after treatment, respectively. The Y-axis shows the Shannon index (alpha-diversity) and the X-axis shows the sample id. Star signed below the patient number means Shannon index change >50%. **B:** The log10 value of relative abundance was calculated and described in different colors. The X-axis

provides the samples' id. The Y-axis provides accumulated genera at different stages (A: in 24 h after hospitalization; C: clinically recovered).

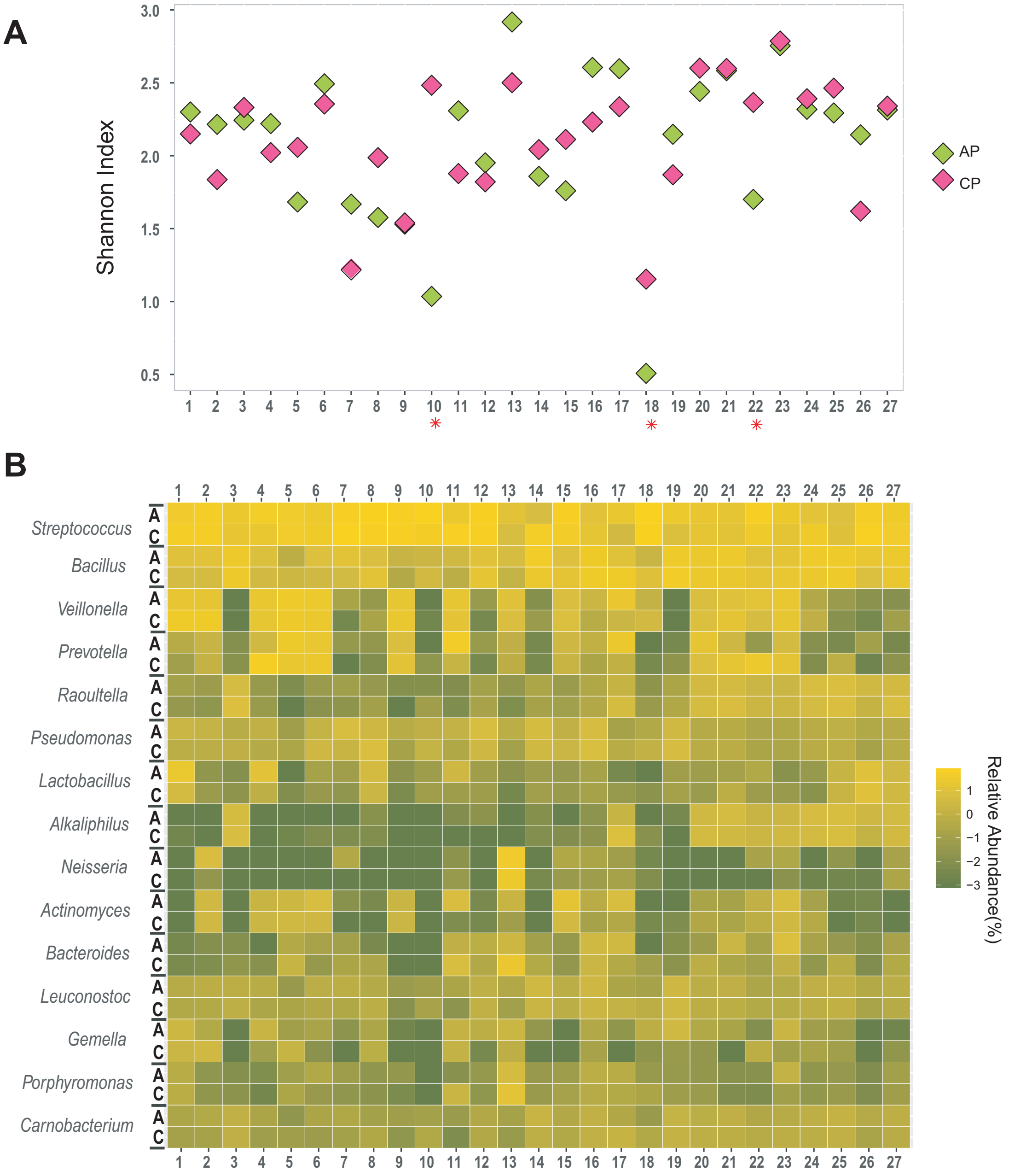

Supplement: Supplementary file 1 — Supplementary Figure [file 41598_2017_11311_MOESM1_ESM.pdf]
